# Transcriptome Analysis of Developing Grains from Wheat Cultivars TAM 111 and TAM 112 Reveal Cultivar-Specific Regulatory Networks

**DOI:** 10.3390/ijms232012660

**Published:** 2022-10-21

**Authors:** Ze-Tian Fang, Rajan Kapoor, Aniruddha Datta, Shuyu Liu, Matthew A. Stull, Paige G. Seitz, Charles D. Johnson, Sakiko Okumoto

**Affiliations:** 1 Department of Soil and Crop Sciences, Texas A&M AgriLife Research, Texas A&M University, College Station, TX 77843, USA; 2 Department of Electrical and Computer Engineering, Texas A&M University, College Station, TX 77843, USA; 3 Texas A&M AgriLife Research Center, 6500 Amarillo Blvd W, Amarillo, TX 79106, USA; 4 Texas A&M AgriLife Genomics and Bioinformatics Service, College Station, TX 77845, USA

**Keywords:** nitrogen, wheat, grains, gene network, transcriptome

## Abstract

Wheat flour’s end-use quality is tightly linked to the quantity and composition of storage proteins in the endosperm. TAM 111 and TAM 112 are two popular cultivars grown in the Southern US Great Plains with significantly different protein content. To investigate regulatory differences, transcriptome data were analyzed from developing grains at early- and mid-filling stages. At the mid-filling stage, TAM 111 preferentially upregulated starch metabolism-related pathways compared to TAM 112, whereas amino acid metabolism and transporter-related pathways were over-represented in TAM 112. Elemental analyses also indicated a higher N percentage in TAM 112 at the mid-filling stage. To explore the regulatory variation, weighted correlation gene network was constructed from publicly available RNAseq datasets to identify the modules differentially regulated in TAM 111 and TAM 112. Further, the potential transcription factors (TFs) regulating those modules were identified using graphical least absolute shrinkage and selection operator (GLASSO). Homologs of the OsNF-Y family members with known starch metabolism-related functions showed higher connectivities in TAM 111. Multiple TFs with high connectivity in TAM 112 had predicted functions associated with ABA response in grain. These results will provide novel targets for breeders to explore and further our understanding in mechanisms regulating grain development.

## 1. Introduction

Wheat (*Triticum aestivum* L.) is an internationally important staple crop whose milled flour is used in many food products ranging from bread to specialty cakes. Due to having different end-use purposes, wheat varieties have been developed according to specific industry standards to achieve desirable qualities. For bread wheat, traits such as high protein content (i.e., percent protein in flour), and protein quality (i.e., gluten quality and ratios of subunits) are thus of high breeding priority aside from grain yield.

The US is projected to have 19.2 million hectares of wheat planted in 2022, of which 71% (i.e., 13.6 million hectares) are projected to be winter wheat [1]. The Southern Great Plain is one of the major winter wheat producing areas, estimated to account for 50% of the total acreage. Hence, retaining and improving high-quality winter wheat varieties adapted to this area is vital to the US wheat and food industry. Hard red winter wheat is used for bread making, for which traits such as percent protein are vital to the success of any released variety. Recent reports on grain crops have shown that grain nitrogen and starch content can be impacted by altering the activities of genes involved in processes such as storage compound biosynthesis or transport of metabolites such as sugar and amino acids [2,3,4], or transcription factors controlling biosynthesis and transport [5,6]. Impactful grain quality genes or candidates are often identified via genetic mapping approaches such as QTL mapping and Genome-Wide Association Study (GWAS). Recent advances in high-throughput sequencing made it possible to identify polymorphisms among cultivars at unprecedented density, contributing to an acceleration of marker-based selection strategies (e.g., genome selection, marker-assisted selection) in breeding programs. However, these techniques that rely on linkage associations lack the ability to identify important regulators that might either lack variation within a limited population (e.g., bi-parental population) or have weaker statistical sensibility due to having a complex population structure, such as being a minor allele or population-specific allele in GWAS. Gene network analysis recently became a method of choice for identifying such candidate regulator genes, mainly due to the ever-increasing availability of high-throughput transcriptome data [7,8,9]. Correlation network among gene expression as well as the studies of causative genes involved in complex phenotypes reveal that genes that have higher interconnectivity within and among the network (often called “hub” genes) are likely to have a large regulatory effect, and therefore a large impact on the phenotypes [10,11,12,13,14]. Thus, utilization of this powerful new tool would aid the effort in identifying central genes crucial to the overall network that can also be potential targets for selection.

In this study, two popular winter wheat varieties (i.e., cv. TAM 111 and cv. TAM 112) were selected due to their superior grain quality, yield, and local popularity. TAM 111 and TAM 112 are genetically related (<40% by pedigree) [15]. Both cultivars are known for their excellent drought resistance ability, and that ability has since been dissected by Reddy et al. and Chu et al. in their transcriptome studies. TAM 112 grains also have a higher percent protein content than TAM 111 at maturity [15,16]. This variation in grain protein content is partly explained by QTLs identified by Dhakal et al. [17]; however, the potential causative mechanism that resulted in the difference in the grain protein content is still unclear. To fill this knowledge gap from a molecular level, we analyzed the transcriptome from grains collected from TAM 111 and TAM 112 at 5 days after flowering (DAF), and 14 DAF representing early and mid-grain developmental stages, respectively. We have identified differentially expressed genes (DEGs) and their related biological pathways via gene-ontology analysis (GO). The GO enrichment suggests that TAM 111 has a developmental emphasis on starch metabolic pathways, whereas TAM 112 seems to be more active in amino acid metabolism and transporter activities in the mid-grain developmental stage. This GO term-based hypothesis was verified via elemental analysis. Lastly, by utilizing graphical least absolute shrinkage and selection operator (GLASSO) in addition to the gene network analysis, we generated a list of potential key regulators that might explain the transcriptomic differences between TAM 111 and TAM 112 during early to middle grain development, which in turn is likely contributing to the final phenotypic variation in grain characteristics.

## 2. Results and Discussion

### 2.1. RNAseq Data Structure

To identify the DEGs between cultivars TAM 111 and TAM 112, RNAseq experiments were performed. Kernels at 5 and 14 days DAF were harvested, representing early and mid-grain filling stages, respectively. The raw reads were mapped to the IWGSC v1.1 wheat genome (Chinese spring, Ensembl, release 49). Approximately, 32 to 40 million reads per sample were mapped to the genome, ranging between 76.9 and 83.2% of total reads mapped, respectively (Table S1).

**Figure 1:**
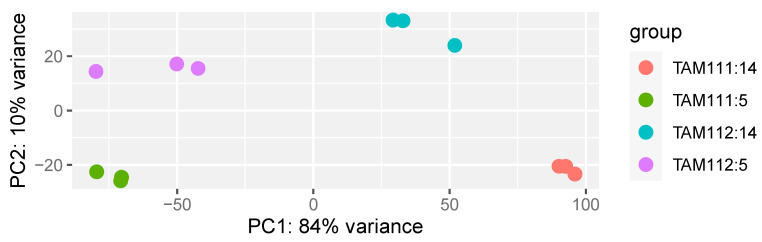
Principle component analysis plot of all samples. Red dots: 14 days after flowering (DAF) samples from TAM111, Green dots: 5 DAF samples from TAM111, teal dots: 14 DAF samples from TAM112, purple dots: 5 DAF samples from TAM112.

A total of 66,502 genes had a significant expression, defined as TPM > 0.5 in at least one sample. In total, 60,416 and 63,952 genes were significantly expressed in 5 and 14 DAF, respectively, among which 58,825 genes were found to be significantly expressed in both datasets. Principle component analysis of variance-normalized read counts of the top 500 most variable genes showed that biological replicates cluster together (Figure 1). The two developmental stages (5 DAF and 14 DAF) separated along the principal component PC1 which accounted for 84% of the variance, whereas PC2 separated the two cultivars, accounting for 10% of the variance. Taken together the result indicated that the effect of the developmental stage (i.e., difference between 5 and 14 DAF) is greater than the genotypic difference (i.e., TAM 111 vs. TAM 112).

### 2.2. DEGs between TAM 111 and TAM 112 at Each Time Points Show Different Pathways Over-Represented

To compare the transcriptome of two cultivars at each of the time points sampled, DEGs were identified. A total of 2629 (1414 and 1215 genes upregulated in TAM 111 and TAM 112, respectively) and 9368 (4868 and 4500 genes upregulated in TAM 111 and TAM 112, respectively) DEGs for 5 and 14 DAF were identified, respectively (Table 1). Comparing the two time points revealed 1937 DEGs as being common at both time points, whereas 692 and 7431 genes were unique to either of the 5 or 14 DAF comparisons, respectively (Table 1). To further explore the variation, DEGs between 5 and 14 DAF within each cultivar were identified using the same criteria described in material and methods. For TAM 111, a total of 14,745 genes (6370 and 8375 genes significantly higher at 5 and 14 DAF, respectively) were developmentally controlled, while in TAM 112 13,061 genes (5481 and 7580 genes significantly higher at 5 and 14 DAF, respectively) were developmentally controlled. Comparing the cultivars transitioning from 5 DAF to 14 DAF, 8044 genes were developmentally regulated in both cultivars, while TAM 111 and TAM 112 each had 6701 and 5017 cultivar-specific DEGs (i.e., total DEGs minus shared DEGs), respectively (Table 1B). The numbers agree with the PCA prediction that the developmental stage caused more significant gene expression changes than cultivar-specific changes at a given developmental stage.

**Figure 2:**
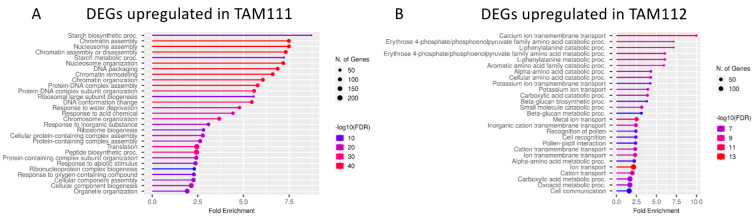
Plots of gene-ontology biological pathway analysis results of genes from 14 DAF expressed higher in TAM111 (**A**) or TAM112 (**B**). Categories (*X*-axis) are sorted by fold enrichment (*Y*-axis). Color presents the relative –log_10_ FDR from low (blue) to high (red). The size of the circle represents the number of genes classified in that category.

Next, to identify the biological pathways over-represented in DEGs between the cultivars and the developmental stages, GO enrichment analysis was conducted using Shiny-GO v0.76 [18]. Comparing the DEGs identified between cultivars at 5 DAF, processes related to DNA structure organization such as chromatin assembly (GO:0031497) and DNA packaging (GO:0006323) were enriched in genes expressed higher in TAM 111 over TAM 112 (Figure S1A), whereas processes related to transports (GO:0070588) and peptidase regulation (GO:0010466) were identified as enriched in the genes expressed higher in TAM 112 over TAM 111 (Figure S1B). At 14 DAF, pathways related to starch biosynthesis (GO:0019252 and GO:0005982) were enriched in the genes expressed higher in TAM 111 compared to TAM 112 (Figure 2A). Processes such as chromatin assembly and other DNA organization processes, over-represented in TAM 111 at 5 DAF-comparison were also enriched in TAM 111 at 14 DAF. In contrast, protein and amino acid metabolic processes (e.g., GO:1902222, GO:0009074, GO:0009063), as well as ion and cellular transport (e.g., GO:0055085 and GO:0006813), were enriched in the genes expressed higher in TAM 112 at 14 DAF (Figure 2B). GO enrichment analyses were also conducted within each cultivar transitioning from 5 to 14 DAF (Figure S2A–D). In TAM 111, when comparing 5 DAF to 14 DAF, genes showing higher expression on 5 DAF are enriched in pathways related to DNA organization (e.g., GO:0006323, GO:0031497, GO:0006334) whereas genes with a higher expression on 14 DAF are enriched in starch synthesis related pathways (e.g., GO:0019252, GO:0005977, GO:0005978). For TAM 112, DEGs that are up-regulated in 5 DAF are highly similar to that of TAM 111, pathways such as DNA organization were highly enriched; however, DEGs up-regulated at 14 DAF are enriched in pathways related to amide (GO:0042886) and peptide transport (GO:0015833), as well as amino acid catabolic processes (GO:0009063). Lastly, common DEGs shared by both cultivars when comparing 5 to 14 DAF were analyzed (Figure S3). Biological pathways expected to be enriched at the mid-filling stage, such as membrane transport (GO:0055085), carbohydrate metabolism (GO:0005975), and cell wall genesis (GO:0044036 and GO:0071554), were detected as well as processes such as plastid transition (GO:0032544) and photosynthesis (GO:0009765). Given that both cultivars showed greening and exhibited a larger seed size at 14 DAF compared to 5 DAF during sample collection, these enrichments supported the physical observation. The overall GO-analysis indicates that the major differences detected between the cultivars at 14 DAF (i.e., TAM 111 being enriched in starch metabolism and TAM 112 being enriched in transporter activities) are also some of the key transitional changes happening from 5 DAF to 14 DAF for the respective cultivars (i.e., starch metabolism and transporter activities are induced at 14 DAF in TAM 111 and TAM 112, respectively), and that transcriptome data pattern is in accordance with the physical observation in the case of shared genes between the two cultivars transitioning from 5 DAF to 14 DAF.

**Figure 3:**
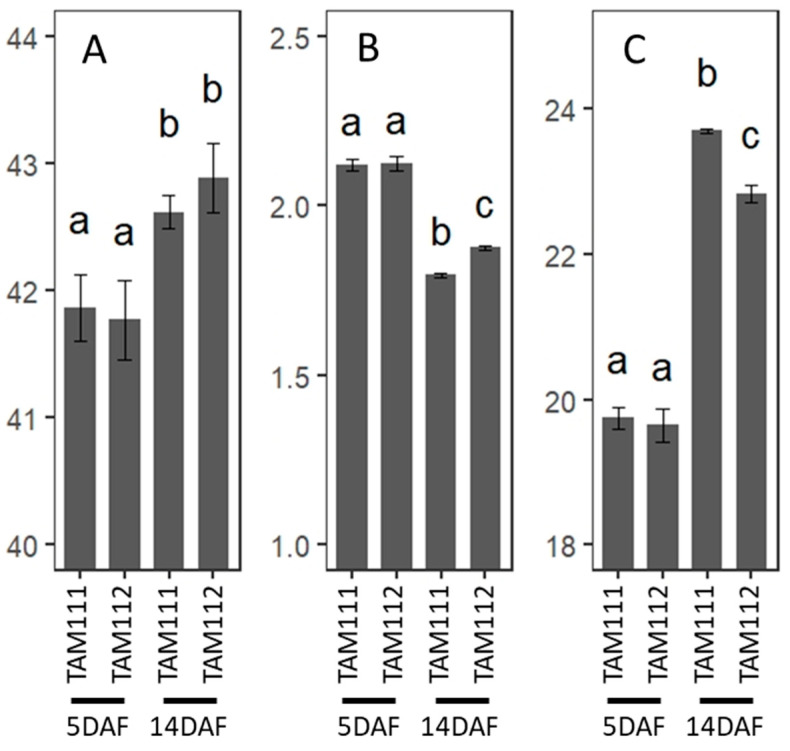
Carbon and nitrogen content of TAM111 and TAM112 grains at 5 and 14 DAF. (**A**) percent carbon, (**B**) percent nitrogen, and (**C**) carbon to nitrogen ratio. Letters indicate significance at α = 0.05 in TukeyHSD-test (n = 3).

Following the trend identified in the GO analysis, we hypothesized that pathways involved in carbon (C)—and nitrogen (N)—containing storage molecules are different between the cultivars; starch synthesis was more active in TAM 111 compared to TAM 112 at 14 DAF whereas genes involved in N transport and metabolism were more active in TAM 112. To test this hypothesis, elemental analysis was conducted using 5 and 14 DAF grains from both cultivars. As expected, carbon content increased significantly from 5 DAF to 14 DAF for both cultivars. The two cultivars are not statistically different for percent carbon at both DAFs (Figure 3A); however, TAM 112 had a significantly higher percent N content at 14 DAF in comparison to TAM 111 (1.80% and 1.88%, respectively, Figure 3B). This higher percentage in grain nitrogen also caused TAM 112 to have a statistically lower C:N ratio versus TAM 111 (23.71:1 vs. 22.84:1, respectively, Figure 3C). These results point to the possible regulatory variation between TAM 111 and TAM 112 regarding nitrogen or amino acid accumulation at 14 DAF. The predicted higher activity of starch synthesis in TAM111 did not seem to raise the C content per dry seed weight. Partitioning of C between the major components (starch, cell walls and free sugars) changes developmentally and varies among the cultivars. For example, previous studies showed that starch:sugar ratio increases dramatically from early- to mid-grain filling, with starch content sharply increasing while the sugar content decreasing [19]. Similarly, the partitioning of C to starch at 14 DAF might be different between TAM 111 and 112.

Taken together, the comparison between the cultivars and time points shows that activities related to grain filling at 14 DAF are different between TAM 111 and TAM 112. In particular, the balance between starch and protein accumulation might be different in TAM 111 and TAM 112, with TAM 112 having a higher capacity for N accumulation. Corroborating with these results from the bioinformatic analysis, the C/N ratio of TAM 111 and TAM 112 grains at 14 DAF was also significantly different at 14 DAF, while 5 DAF was not.

### 2.3. Gene Network Analysis to Identify Modules Enriched in DEGs

The results above suggest differences in pathways activated during grain filling of TAM 111 and TAM 112. With the goal of identifying regulatory elements responsible for genotype-specific expression patterns, we first identified the DEGs belonging to the biological pathways enriched in the genotypes (e.g., starch synthesis in TAM 111). However, DEGs do not generate a comprehensive list of possible associated genes within a pathway and thus might limit or bias the analysis. Therefore, we reasoned that by overlaying the expression patterns found from our study onto the gene networks obtained using publicly available grain expression data from multiple cultivars, we will be able to capture additional genes that are generally coregulated with the DEGs found in our study. In other words, this process will put the DEGs in the context of larger regulatory networks activated during the grain development that are shared among different cultivars. Additionally, using additional datasets improves the accuracy of the network analysis [19,20].

**Figure 4:**
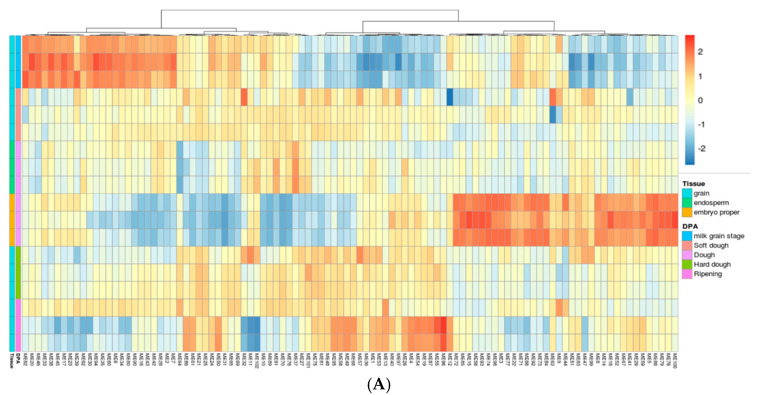

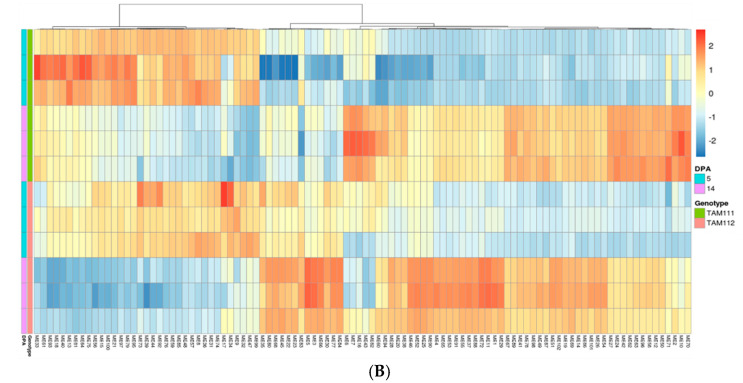
Gene modules constructed from WGCNA using publicly available grain RNAseq data. (**A**): Expression pattern of eigengenes representing each module. Modules are clustered by tissue type and developmental stages (colored rectangles on the left). (**B**): Expression pattern of module genes in TAM111 and TAM112. Modules are clustered by genotype and DPA, indicated by colored rectangles on the left. Hierarchical clustering has been performed to identify modules with similar expression patterns.

To construct a reliable gene network in developing wheat grains, publicly available RNAseq data from non-stressed grain tissues (n = 118) was used for Weighted Gene Correlation Network Analysis (WGCNA) [20,21]. The network accounted for batch (i.e., samples from each study) variation but not for the developmental stage (i.e., DAF), and thus the resulting modules were representative of biological activities correlated not only to gene expression similarity but also to the developmental stages. A total of 102 modules were detected, to which 52,506 genes were assigned (Table S2). The resulting modules clustered according to the expression patterns among tissue types (i.e., whole grain, endosperm, and embryo) and developmental stages (e.g., milk grain stage, soft dough, dough, etc.) as expected (Figure 4A). To identify the modules that have significant variation between TAM 111 and TAM 112, the expression of genes belonging to each module identified in the WGCNA analysis above were used to calculate the eigengene that represents the overall trend of module expression in TAM 111 and TAM 112 (Figure 4B). By visualizing the relative expression shifts, major clusters of modules could be identified where either cultivar (TAM 111 or TAM 112) shows a significantly higher expression compared to the other. Focusing on 14 DAF, at which stage we observed N content difference between the cultivars (Figure 3), we identified five clusters of modules (three were termed TAM 111 A–C, and the other two termed TAM 112 A and B) (Figure S4).

To identify the biological significance of these groups of modules, genes belonging to the modules labeled TAM 111A-C and TAM 112A-B were extracted and then analyzed individually using Shiny-GO. TAM 111 region A showed significant enrichment in pathways related to DNA organization (e.g., GO:0006334, GO:0031497, GO:0034728, etc. Figure S5A), while region B showed significant enrichment in starch metabolism (GO:0005982) related pathways such as amylopectin (GO:0010021 and GO:2000896) and glycogen metabolism (GO:0005978 and GO:0005977) (Figure S5B). Interestingly, region C had enrichments in stress-related pathways such as heat-stress (GO:0009408) and response to hydrogen peroxide (GO:0042542) as well as regulation of peptidase-related pathways (GO:0052547 and GO:0010466, Figure S5C). TAM 112 region A was enriched in pathways related to phosphatidylinositol (GO:0046488) and glycerophospholipid (GO:0006650) metabolism, DNA repair (GO:0006281), and intracellular transport (GO:0046907), whereas region B was enriched in pathways related to protein transport and localization (GO:0015031, GO:0033365, GO:0034613, etc.), amide transport (GO:0042886), as well as intracellular transport (GO:0046907) (Figure S5D–E). These data agree with the pathways identified with the DEG analysis; groups such as TAM 111A, TAM 111B, and TAM 112 B are highly enriched in biological pathways that were identified to be more active in the respective cultivar (Figure 2). This indicates that the gene network constructed adequately described and clustered genes, and was capable of reflecting shifts in biological activities specific to TAM 111 and TAM 112.

### 2.4. Transcription Factors Identified by GLASSO to Be Central to Starch Synthesis

Transcription factors have been identified as central regulators for grain traits in other grain crops, such as rice and maize [22,23,24]. Thus, to identify candidate TFs that are responsible for the difference in networks between TAM 111 and TAM 112, GLASSO was implemented to identify TFs that show high connectivity specifically in one of the two cultivars. To do this, the genes belonging to each individual group that was differentially regulated among the two cultivars (i.e., TAM 111 A-C and TAM 112 A and B, Figure S4) were combined with the list of high-confidence TFs from the wheat genome [21,25] and was used for conducting the GLASSO analysis. The resulting networks specific to TAM 111 vs. TAM 112 were compared, and the genes were ranked according to the absolute difference in the number of edges between the two networks. The genes with higher connectivity in TAM 111 indicate that the top genes were highly consistent (e.g., TracesCS7B02G248300, TraesCS7D02G344400, and TraesCS5A02G245900) regardless of which subset we used (Table 2A–C). The genes with higher connectivity in TAM 112 network also had some commonly shared genes (e.g., TraesCS4A02G231300 and TraesCS4A02G131000) with high connectivity in both regions (Table 3A,B).

Notably, three homeologs of TFs belonging to Nuclear Factor-Y (NF-Y) family (TraesCS7A02G336700, TraesCS7B02G248300, and TraesCS7D02G344400) consistently ranked as highly connected in TAM 111 compared to TAM 112, and are the closest homolog to OsYC11 and OsYC12 (Figure S6). Previous studies have shown that OsNF-YC11 and 12 are associated with OsNF-YB1 and OsbHLH144 to regulate grain filling and endosperm development [26]. OsNF-YC complex was described to directly bind to the starch synthesis genes in rice in developing grains and that OsbHLH144 determines the complex stability of OsNF-YC [23]. A similar triad between NF-YB, NF-YC and bHLH genes might be involved in the upregulation of starch synthesis, and might be more active in TAM 111. In our RNA data set, one of the three NF-YC genes (TraesCS7A02G336700) was differentially expressed between TAM 111 and TAM 112. Whether the variation in the activities of starch biosysnthesis genes observed in TAM 111 and TAM 112 was influenced by a pathway involving NF-YC, NF-YB, and bHLH system described in rice remains to be seen.

In TAM 112, there are also several TFs shared between the two regions, especially the homeodomain-leucine zipper (HD-Zip) TF family. TraesCS4A02G231300 (TaHDZ30A), TraesCS3A02G325800/TraesCS3B02G354900 (TaHDZ29A and B), and TraesCS2A02G389400 (TaHDZ7A) belong to the HD-Zip TF family’s clade IV (δ), IV (ζ), and I (γ), respectively [27]. Many of the plant HD-Zip transcription factors characterized so far are involved in developmental processes and environmental stress responses, especially drought stress through ABA signaling [28,29,30,31,32]. High connectivity of HD-Zip in our dataset seems to suggest that some HD-Zip members might also be a part of the regular grain development process associated with ABA or other hormones. The GLASSO analysis of TAM 112-A also identified three homeologs of TaDREB5 (i.e., Traes1A02G244800/TraesCS1B02G256000/TraesCS1D02G244500). TaDREB5 is induced by drought and ABA, and its close homolog TaDREB3 improves drought resistance in overexpression lines in Arabidopsis [33]. Previous work showed that TAM 112 consistently shows higher ABA responses not only in drought environments but also during regular development in comparison to TAM 111 [15,16]. Hence, the differential connectivity for ABA- and drought-responsive TFs between TAM 111 and TAM 112 seems to corroborate the previous results. Further investigation is required to examine whether the differential response to ABA between the cultivars underlines the grain phenotype differences between the cultivars.

## 3. Materials and Methods

### 3.1. Plant Materials

Wheat (Triticum aestivum cv. TAM 111 and TAM 112) plants were grown under field condition described in our previous experiment [34]. Grains from 5 Days after flowering (DAF), and 14 DAF were collected and stored in −80 °C for total RNA extraction.

### 3.2. RNA Extraction and Sequencing

Total RNA extraction was performed using a modified method from Li and Trick [35], then cleaned using Qiagen^®^ (Germantown, MD, USA) RNeasy Plant Kit. Each sample consists of total RNA extract from several similar sized developing grains collected from the same inflorescence. The processed total RNA was sent to Genomic and Bioinformatics Service, Texas A&M AgriLife, Texas A&M University for poly-A selection, cDNA library construction, and sequencing. mRNA was isolated using a Nextflex^®^ Poly-A Selection kit (Perkin Elmer, Waltham, MA, USA). cDNA libraries were generated using a Nextflex^®^ Rapid Directional RNA kit. The libraries of three highest quality samples per cultivar (i.e., TAM 111 and TAM 112) per DAF (i.e., 5 DAF and 14 DAF), a total of 12 separate samples were sequenced on a NovaSeq 6000 using an S4 flow cell with 100 nucleotide paired-end reads (Illumina, San Diego, CA, USA), approximately 50 million paired reads per sample.

### 3.3. RNAseq Data Processing

Initial quality assessment was monitored via FASTQC tool (0.11.9-Java-11) [36]. Adapters were trimmed via Cutadapt (2.8-GCCcore-8.3.0-Python-3.7.4) [37], then quality trimming was performed using Trimmomatic (0.39-Java-11) in pair-end mode (LEADING:5, TRAILING:5, SLIDINGWINDOW:4:15, MINLEN:30) [38]. The resulting PE and SE files were mapped using HISAT2 (2.1.0-intel-2017b) against the wheat genome (IWGSC RefSeq assembly v1.0) [39], then sorted by SAMtools (1.9-intel-2018b) into BAM files [40]. Raw counts were produced by Subread (1.6.2-Linux-x86_64) using function “FeatureCounts” with the wheat genome annotation (IWGSC RefSeq annotation v1.1) [41].

### 3.4. Differential Expressed Genes (DEG) and Gene Ontology Enrichment (GO) Analysis

The raw count data mentioned above was fed into R package and analyzed using the following factored design to account for genotype-specific development effects: ~genotype + day + genotype:day, with TAM 111 and day 5 selected as reference factors [42]. The analysis was to compare between cultivars on DAF 5 and DAF 14 (i.e., TAM 111 vs. TAM 112, at 5 DAF and 14 DAF), then within cultivar from 5 DAF to 14 DAF (i.e., TAM 111 5 DAF vs. 14 DAF, and TAM 112 5 DAF vs. 14 DAF). DEG were declared based on the criteria of having an absolute value of log_2_Foldchange > 1 corrected by R-package ‘ashr’ and adjusted *p*-value < 0.01 corrected by R-package ‘IHW’ [43]. GO analysis was performed by using ShinyGO v.0.76 [18] with an FDR cutoff of 0.05 and minimum pathway size of 10 and maximum size of 2000.

### 3.5. Constructing Gene Network Analysis

In order to establish the networks of co-expressed genes in grains, publicly available RNAseq data sets from non-stressed grain tissues (n = 118) [20,21] were quasi-mapped to the gene models (IWGSC v1.1). The list of datasets used can be found in Supplemental Table S3. High confidence genes with TPM expression >0.5 in at least one sample were selected to construct the network. Variance stabilizing transformation (vst) from DESeq2 was used to minimize the effects of sequencing depth between studies. Batch effects were first removed using ‘removeBatchEffect’ function from the R-package ‘Limma’ [44], then the effects of variables other than developmental stage and grain layer were removed using ‘ComBat’ function from the ‘sva’ package. Next, signed hybrid networks were constructed using WGCNA [45]. Maximum block size of 95,000 genes was used to cover all high confidence genes in a single block for maximum accuracy of module assignment. The modules with more than 85% similarity were merged using parameter mergeCutHeight = 0.15, resulting in a total of 102 modules which contained 52,506 unique genes. Lastly, the hierarchical clustering was improved using the ‘km2gcn’ package [46].

### 3.6. GLASSO Analysis

The clusters of modules identified using WGCNA were used to create gene network graphs using function ‘fgl’ from R package ‘JGL’ [47] with penalties: lambda1 = 1 and lambda2 = 0 to focus only on differential edges between TAM 111 and TAM 112. The data was variance stabilized using function ‘rlog’ from DESeq2 before using it for GLASSO analysis. Full results of GLASSO analyses are presented in Supplemental Tables S4–S8.

### 3.7. Phytogenic Tree Analysis of NF-YC and NF-YB

Wheat NF-YC and YB genes have been mined from IWGSC RefSeq annotation v1.1 using protein BLAST, with an e-value cutoff at 1 × 10^−4^. Protein sequences were aligned with MUSCLE program [48], with the UPGMA (unweighted pair group method with arithmetic mean) method and 100 iterations, gap open penalty −2.9, extend penalty 0, and hydrophobicity multiplier 1.2. The alignment was used to build a phylogenetic tree using MEGA X program [49], using maximum likelihood method with the nearest-neighbor-Interchange inference option and the bootstrap value of 1000.

### 3.8. C and N Analysis of Wheat Grains

Wheat grains at 5 and 14 DAF were lyophilized and milled. The total C and N content was measured in a total combustion elemental analyzer (Vario EL Cube, Elementar Americas Inc., Ronkonkoma, NY, USA).

## 4. Conclusions

In this study, we have utilized the transcriptome data to identify two separate sets of hub TFs with consistently high connectivity in the respective, TAM 111 and TAM 112 modules of genes. The TFs associated with TAM 111 are highly homologous to the known TFs involved in starch synthesis activation in rice, while TAM 112-upregulated TFs are related to ABA and other hormone-associated biological pathways. The changes in either pathway offer potential to influence grain protein content either directly by upregulating amino acid metabolism and transport, or indirectly by influencing the starch content and therefore grain weight and relative protein percentage, as previously documented [50]. Thus our study has provided a list of potential targets for breeders to explore and leads for future studies that would verify their binding as well as their specific impact on grain development.

## Figures and Tables

**Table 1:** DEG statistics of TAM111 vs. TAM112 (**A**) and 5 DAF vs. 14 DAF (**B**). DEGs were identified using the criteria |−log_2_| > 1, and Adj. *p* < 0.01. The numbers indicate the number of genes identified. Shared row indicates the numbers of identical DEGs between the two time points (top) or the cultivars (bottom).

A. DEG Analysis of TAM111 vs. TAM112			
	Total DEGs	Upregulated in TAM111	Upregulated in TAM112
5 DAF	2629	1414	1215
14 DAF	9368	4868	4500
Shared	1937	1081	856
**B. DEG Analysis of 5 vs. 14 DAF**			
	**Total DEGs**	**Upregulated in 5 DAF**	**Upregulated in 14 DAF**
In TAM111	14,745	6370	8375
In TAM112	13,061	5481	7580
Shared	8044	3248	4754

**Table 2:** Top 10 transcription factors identified from GLASSO using the module groups expressed higher in TAM111. (**A**–**C**) represents regions from TAM111 shown in Figure S4. Lists are sorted by the difference in the number of edges between the two cultivars.

A. GLASSO TAM111A				
Gene	Transcription Factor Family	Edges in TAM111	Edges in TAM112	Difference
TraesCS7B02G248300	Nuclear Factor-Y	208	36	172
TraesCS5B02G043100	MYB-related	39	0	39
TraesCS5A02G245900	NAC	48	20	28
TraesCS7D02G196300	NAC	35	12	23
TraesCS7B02G056300	NAC	33	12	21
TraesCS7A02G336700	Nuclear Factor-Y	46	26	20
TraesCS3A02G368700	MYB-related	23	3	20
TraesCS7B02G100300	NAC	37	19	18
TraesCS7D02G344400	Nuclear Factor-Y	58	41	17
TraesCS5D02G161000	C2C2_Dof	26	9	17
**B. GLASSO TAM111B**				
**Gene**	**Transcription Factor Family**	**Edges in TAM111**	**Edges in TAM112**	**Difference**
TraesCS7B02G248300	Nuclear Factor-Y	343	16	327
TraesCS7D02G344400	Nuclear Factor-Y	125	16	109
TraesCS7A02G336700	Nuclear Factor-Y	97	9	88
TraesCS5B02G043100	MYB-related	68	0	68
TraesCS7D02G216600	CCAAT_Dr1	36	9	27
TraesCS5A02G155900	C2C2_Dof	40	21	19
TraesCS2D02G234000	MYB-related	22	5	17
TraesCS7A02G194700	NAC	55	39	16
TraesCS5B02G154100	C2C2_Dof	35	19	16
TraesCS4B02G299000	MYB-related	18	2	16
**C. GLASSO TAM111C**				
**Gene**	**Transcription Factor Family**	**Edges in TAM111**	**Edges in TAM112**	**Difference**
TraesCS7B02G248300	Nuclear Factor-Y	448	39	409
TraesCS5B02G043100	MYB-related	80	0	80
TraesCS5A02G245900	NAC	43	8	35
TraesCS3A02G077900	NAC	35	6	29
TraesCS7D02G154200	NAC	34	8	26
TraesCS7B02G056300	NAC	32	10	22
TraesCS7A02G569300	NAC	23	1	22
TraesCS7B02G489500	NAC	30	10	20
TraesCS7D02G344400	Nuclear Factor-Y	69	52	17
TraesCS7A02G336700	Nuclear Factor-Y	47	30	17

**Table 3:** (**A**,**B**) Top 10 transcription factors identified from GLASSO using the module groups expressed higher in TAM112. (**A**,**B**) represents regions from TAM112 in Figure S4. Lists are sorted by the difference in the number of edges between the two cultivars.

A. GLASSO TAM112A				
Gene	Transcription Factor Family	Edges in TAM111	Edges in TAM112	Difference
TraesCS4A02G231300	HD-Zip_IV	96	2325	2229
TraesCS1B02G256000	AP2/EREBP	63	258	195
TraesCS3A02G325800	HD-Zip_IV	3	214	211
TraesCS2A02G389400	HD-Zip_I_II	2	172	170
TraesCS4A02G131000	NAC	11	140	129
TraesCS1D02G244500	AP2/EREBP	60	116	56
TraesCS1A02G244800	AP2/EREBP	60	109	49
TraesCS3B02G354900	HD-Zip_IV	3	97	94
TraesCS7B02G112400	MYB-related	68	88	20
TraesCS4A02G263300	bHLH	51	85	34
**B. GLASSO TAM112B**				
**Gene**	**Transcription Factor Family**	**Edges in TAM111**	**Edges in TAM112**	**Difference**
TraesCS4A02G231300	HD-Zip_IV	104	253	149
TraesCS2A02G389400	HD-Zip_I_II	3	68	65
TraesCS5A02G155900	C2C2_Dof	31	83	52
TraesCS4A02G131000	NAC	10	60	50
TraesCS3A02G325800	HD-Zip_IV	3	52	49
TraesCS7A02G189200	NAC	20	55	35
TraesCS2B02G119100	NAC	4	39	35
TraesCS3B02G354900	HD-Zip_IV	1	36	35
TraesCS4A02G006100	MYB-related	13	46	33
TraesCS4D02G051600	bHLH	25	52	27

## Data Availability

The raw RNAseq data and aligned. bam files are found in GEO (accession number GSE210495).

## Supplementary Materials

The supporting information can be downloaded at: https://www.mdpi.com/article/10.3390/ijms232012660/s1.
