# Supplementary material for: Transcriptome Analysis of Developing Grains from Wheat Cultivars TAM 111 and TAM 112 Reveal Cultivar-Specific Regulatory Networks
Source: Int J Mol Sci. 2022 Oct 21;23(20):12660. doi: 10.3390/ijms232012660 (PMC9604430; doi:10.3390/ijms232012660)
Supplement: Supplementary file 1 [file ijms-23-12660-s001.zip › Supplemental_figures.pdf]

A DEGs upregulated in TAM111

B DEGs upregulated in TAM112

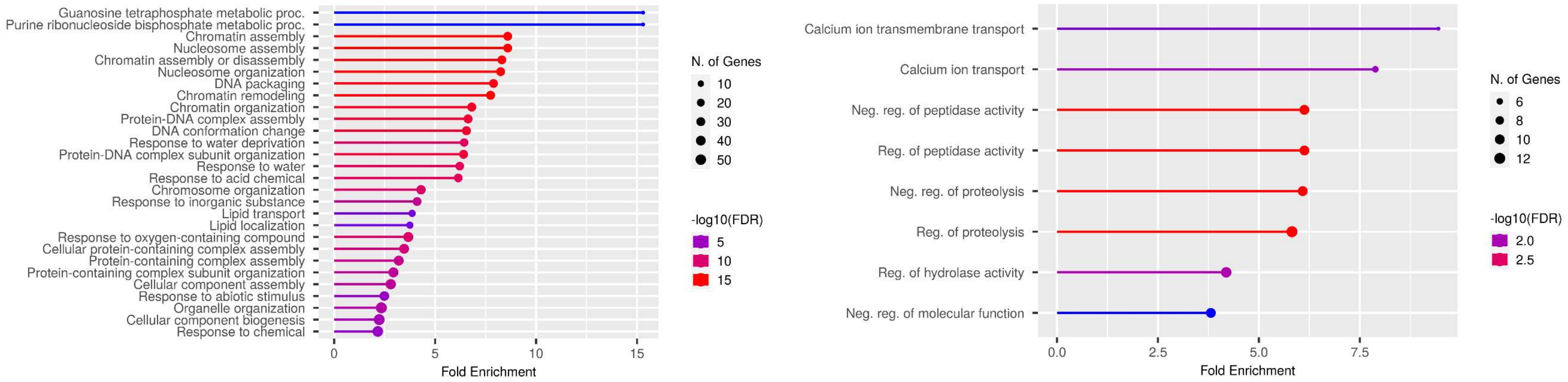

Supplemental Figures S1A and B. Plots of gene-ontology biological pathway analysis results of genes from 5DAF expressed higher in TAM111(A) or TAM112(B)..

Categories are sorted by fold enrichment. Bar colors present the relative $-\log_{10}$ false discovery rate (FDR). The size of the circle represents the number of genes classified in that category.

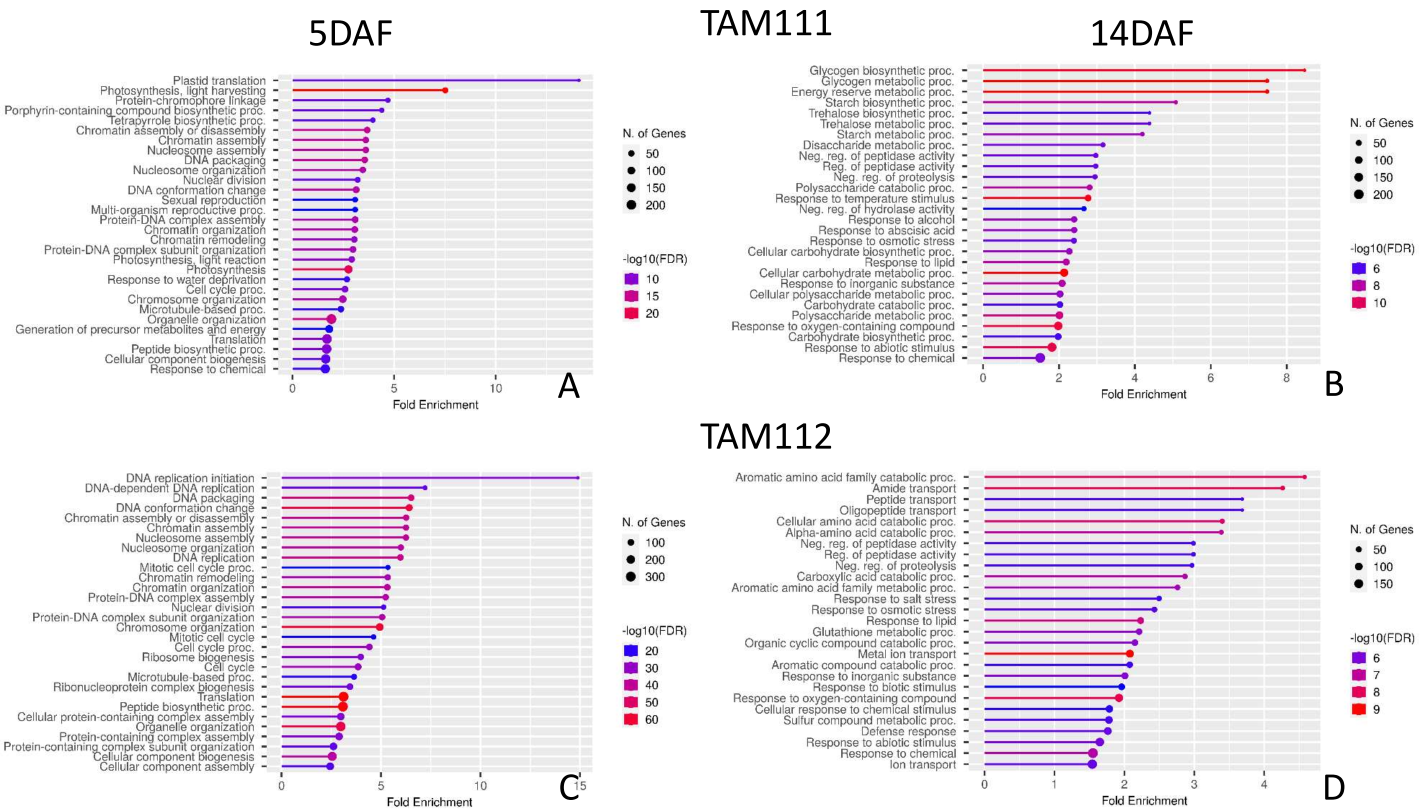

Supplemental Figures S2A-D. Plots of gene-ontology biological pathway analysis results of DEGs from 5DAF vs 14 DAF in TAM111 and TAM112. Categories (X-axis) are sorted by fold enrichment (Y-axis). Color presents the relative $-\log_{10}$ FDR from low (blue) to high (red). The size of the circle represents the number of genes classified in that category.

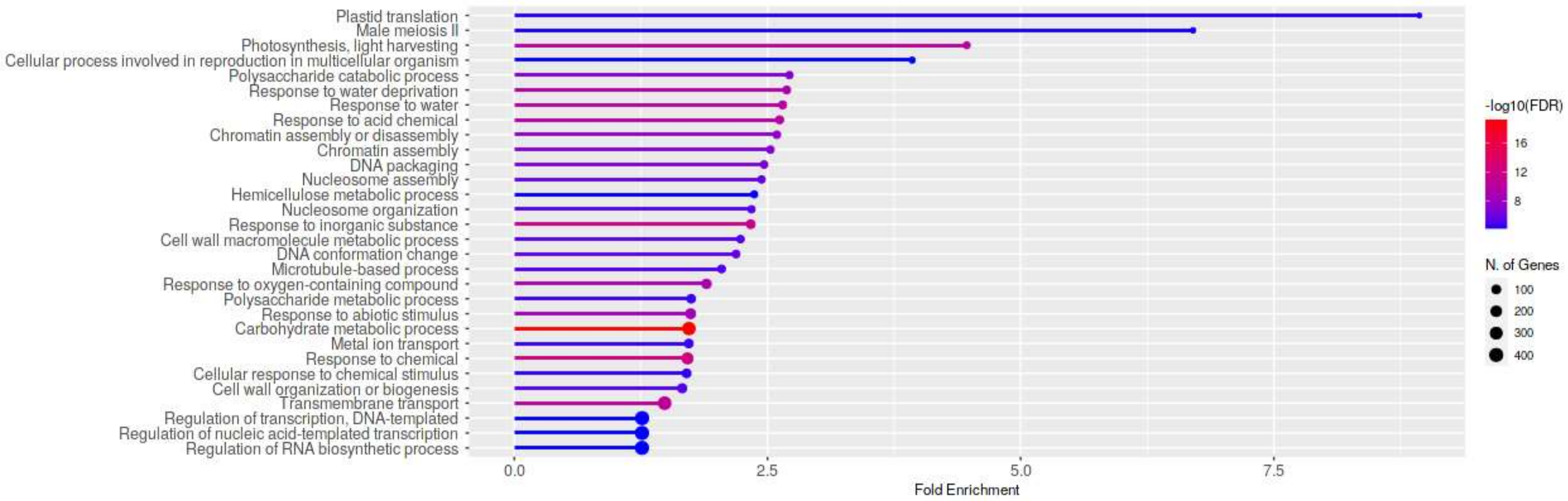

Supplemental Figure S3. Plots of gene-ontology biological pathway analysis results of common DEGs from 5DAF vs 14 DAF in TAM111 and TAM112.

Categories (X-axis) are sorted by fold enrichment (Y-axis). Color presents the relative $–\log_{10}$ FDR from low (blue) to high (red). The size of the circle represents the number of genes classified in that category.

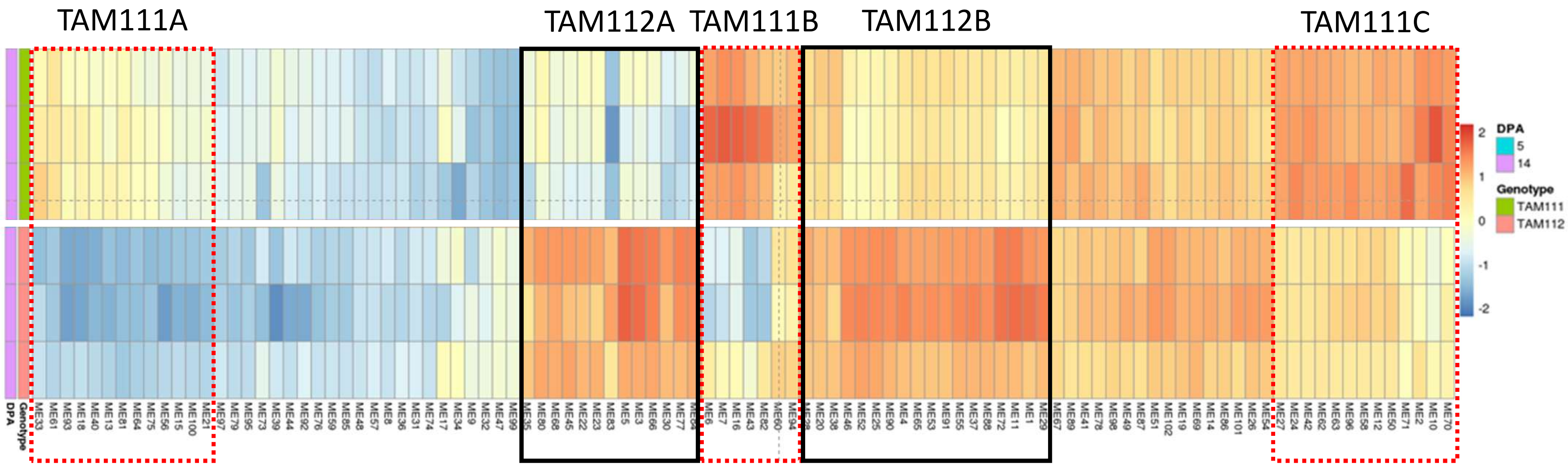

Supplemental Figure S4. Groups of modules differentially regulated between the two cultivars at 14 DAF. Red dotted lines indicate the regions of relative upregulation in TAM111 vs TAM112 at 14DAF.

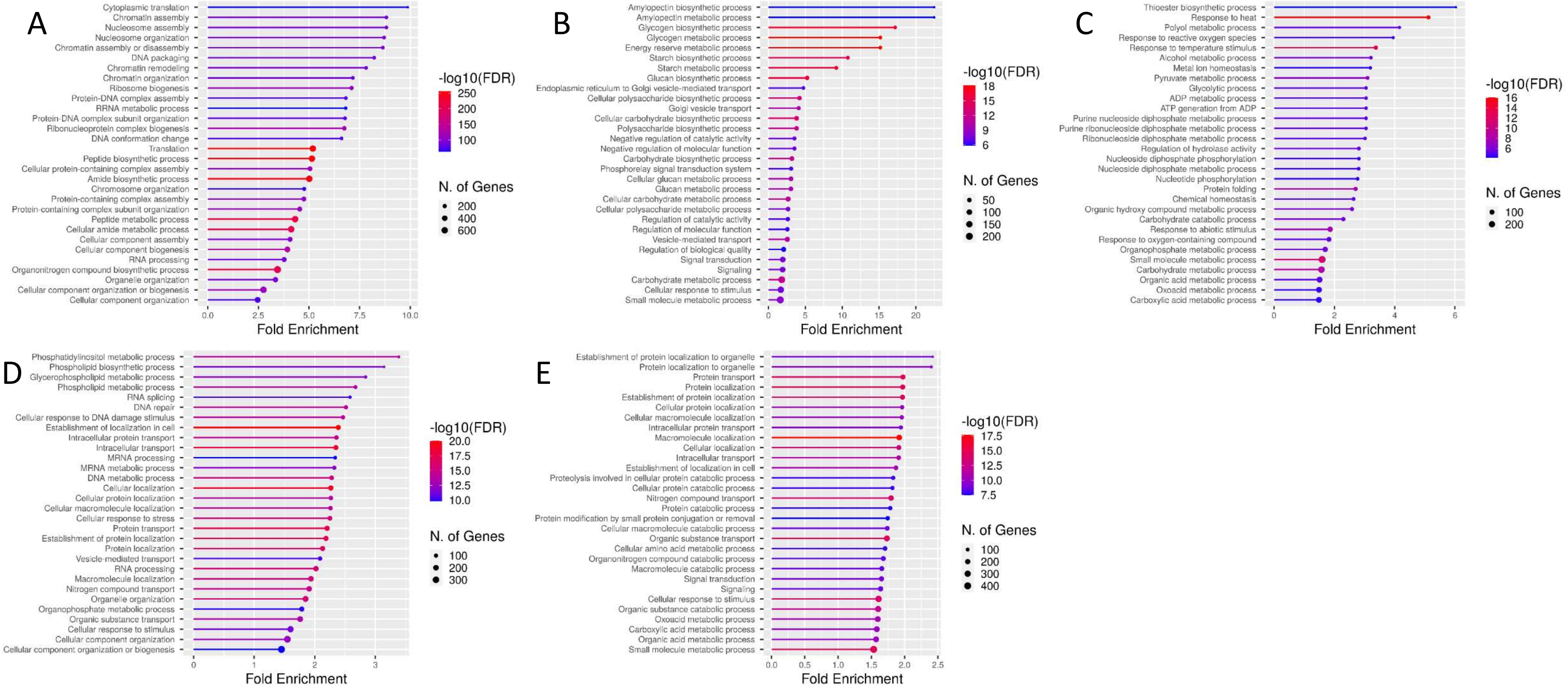

Supplemental Figures S5A-E. Plots of gene-ontology biological pathway analysis results of genes from regions identified in WGCNA (Fig. 3S). A-C represents TAM111
region A-C. D and E represents TAM112 region A and B. Categories (X-axis) are sorted by fold enrichment (Y-axis). Color presents the relative $-\log_{10}$ FDR from low (blue) to high (red). The size of the circle represents the number of genes classified in that category.

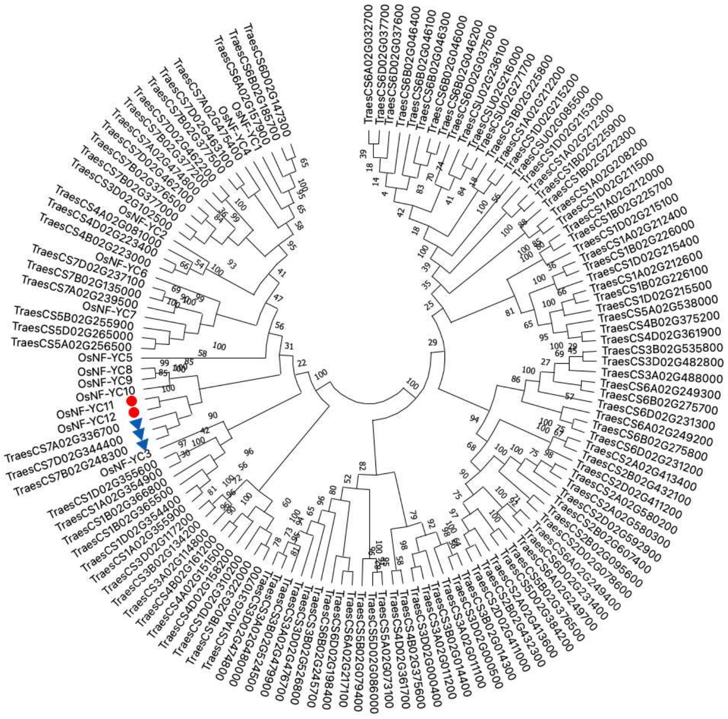

Supplemental Figure S6. A phytogenic tree of NF-YC family genes from wheat and rice. OsYC11 and YC12, and their closest homologs in wheat are labeled with red circles and blue triangles, respectively.
